# Emerging Applications of Liquid Crystals Based on Nanotechnology

**DOI:** 10.3390/ma7032044

**Published:** 2014-03-11

**Authors:** Jung Inn Sohn, Woong-Ki Hong, Su Seok Choi, Harry J. Coles, Mark E. Welland, Seung Nam Cha, Jong Min Kim

**Affiliations:** 1 Department of Engineering Science, University of Oxford, Oxford OX1 3PJ, UK; E-Mail: jong.kim@eng.ox.ac.uk; 2 Jeonju Center, Korea Basic Science Institute, Jeonju, Jeollabuk-do 561-180, Korea; E-Mail: wkh27@kbsi.re.kr; 3 Centre of Molecular Materials for Photonics and Electronics, Department of Engineering, University of Cambridge, Cambridge CB3 0FA, UK; E-Mails: suseok.choi@gmail.com (S.S.C.); hjc37@cam.ac.uk (H.J.C.); 4 Nanoscience Centre, University of Cambridge, Cambridge CB3 0FF, UK; E-Mail: mew10@cam.ac.uk

**Keywords:** liquid crystal, transistor, holography, display, energy harvesting

## Abstract

Diverse functionalities of liquid crystals (LCs) offer enormous opportunities for their potential use in advanced mobile and smart displays, as well as novel non-display applications. Here, we present snapshots of the research carried out on emerging applications of LCs ranging from electronics to holography and self-powered systems. In addition, we will show our recent results focused on the development of new LC applications, such as programmable transistors, a transparent and active-type two-dimensional optical array and self-powered display systems based on LCs, and will briefly discuss their novel concepts and basic operating principles. Our research will give insights not only into comprehensively understanding technical and scientific applications of LCs, but also developing new discoveries of other LC-based devices.

## 1. Introduction

Liquid crystals (LCs), first discovered in 1888 by an Austrian botanist, Frederick Reinitzer, continue to attract intense research interest because of their orientational order, the existence of strong dipoles and easily polarizable groups, the rigidness of the long axis, anisotropic features in the structural, optical, electrical and magnetic properties, as well as their easy response to electric, magnetic and surface forces [1–5].

Much effort has been devoted to the great scientific and technological developments and achievements for advanced photonic devices and high-performance display applications of LCs, as well as for creating novel device concepts and new LC applications. As a result, LCs will be able to become ubiquitous in diverse applications, ranging from displays to electronics, sensors, lasers and optical computing in our daily life [2,6].

To date, besides the familiar displays being the most common applications of LC technology, numerous studies on LCs have been performed to provide unparalleled opportunities to facilitate the basic understanding of the science and to develop new non-display applications. In this regard, especially, ferroelectric LCs that possess a variety of advantages, such as a permanent electric polarization, high flexibility and fast response time, have attracted considerable attention in recent years as the basis for noble field effect transistors (FETs), memory cells and optical switching device applications [5,7]. In particular, among FET-based non-volatile memory devices, the ferroelectric FET, an important type of memory cells without a storage capacitor, is significantly attractive for memory and switch applications, because of the wide range of its interesting features, including small cell size, non-destructive read-out, low-power consumption, good retention and fast response time [8–15]. Another example of emerging applications based on LCs is holography, which is an ideal technology to realize three-dimensional (3D) dynamic images by changing the refractive index [16,17]. However, unfortunately, photo-refractive materials and photo-chromic materials require high external voltages and long response times, respectively, to modulate the refractive index [18–22]. To avoid such difficulties, arrays of carbon nanotubes (CNTs)/nanofibers were introduced to locally modulate the refractive index of the LC medium at low operation voltages [23–25]. Recently, we also developed a transparent, active-type 2D optical array that operates at low voltage using a graphene/CNT hybrid structure instead of using opaque metals [16]. In addition to demonstrating proof-of-principle concepts for individual single LC device applications, it is also possible to achieve technological advances and revolution into a rapidly growing multidisciplinary field involving displays, electronics and energy harvesting to integrate displays into a self-powered system [26,27]. Recent developments in piezoelectric power generators harvesting energy steadily from ambient mechanical vibrations without regard to time, place or any external conditions, present innovative and emerging research topics [26,28–32]. Among various piezoelectric materials and structures, a ZnO nanowire has been intensively studied as one of the most attractive materials. In particular, recent advance in the ZnO nanowire-based piezoelectric power generators exhibited highly promising piezoelectric performance for a self-powered source, due to its dimensionality and piezoelectric semiconducting properties containing non-toxicity, eco-friendliness and geometrical versatility [28,30,33].

In this paper, we aim to present and review snapshots of our recent research carried out on emerging applications of LCs, particularly focusing on the development of new applications based on a combination of LCs and functional nanomaterials, such as, ZnO nanowires, CNTs and graphene. In addition, we will briefly discuss novel device concepts and basic principles of programmable transistors, a transparent and active-type 2D optical array and multifunctional hybrid systems based on LCs. These explorations will give insights not only in comprehensively understanding the technical and scientific applications of LCs, but also developing new discoveries of other LC-based devices.

## 2. Results and Discussion

### 2.1. Programmable Transistors Based on Ferroelectric Liquid Crystals

**Figure 1.**
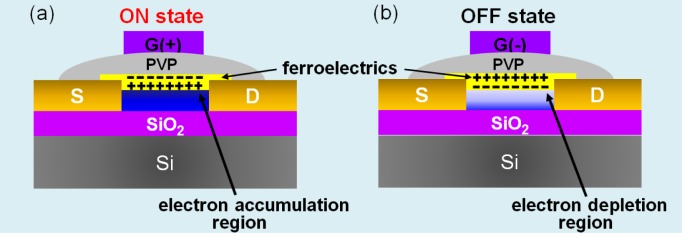
Schematic views of ferroelectric liquid crystal (LC)-coated ZnO nanowire field effect transistors (FETs) exhibiting charge accumulation and depletion in the channel. (**a**) The ON memory state; (**b**) the OFF memory state. PVP, poly(4-vinylphenol).

The intrinsic memory functionality of ferroelectric FETs based on a semiconducting channel and ferroelectric materials arises from the modulation of the charge carrier concentration in the channel, which defines two different conductance states, by switching the polarization of the ferroelectrics electrically, as shown in Figure 1. When a positive gate pulse is applied, the polarization of ferroelectrics is rearranged in the direction of the ferroelectric-semiconductor interface, resulting in the build-up of positive charges on the semiconducting surface. Subsequently, this leads to the accumulation of electrons and, thus, a high conductance state, corresponding to the ON state (Figure 1a) [7,12]. Conversely, when a negative gate pulse is applied, the negative polarization leads to the depletion of electrons and results in a low conductance state, that is, the OFF state (Figure 1b). Accordingly, to create novel functionality in FETs using ferroelectric materials as a new approach for the fabrication of programmable ferroelectric transistors, serving as a hybrid cell for memory, switch and display applications [34–37], we must first demonstrate ferroelectric LC-induced conductance modulation and hysteresis behaviour in a FET.

**Figure 2.**
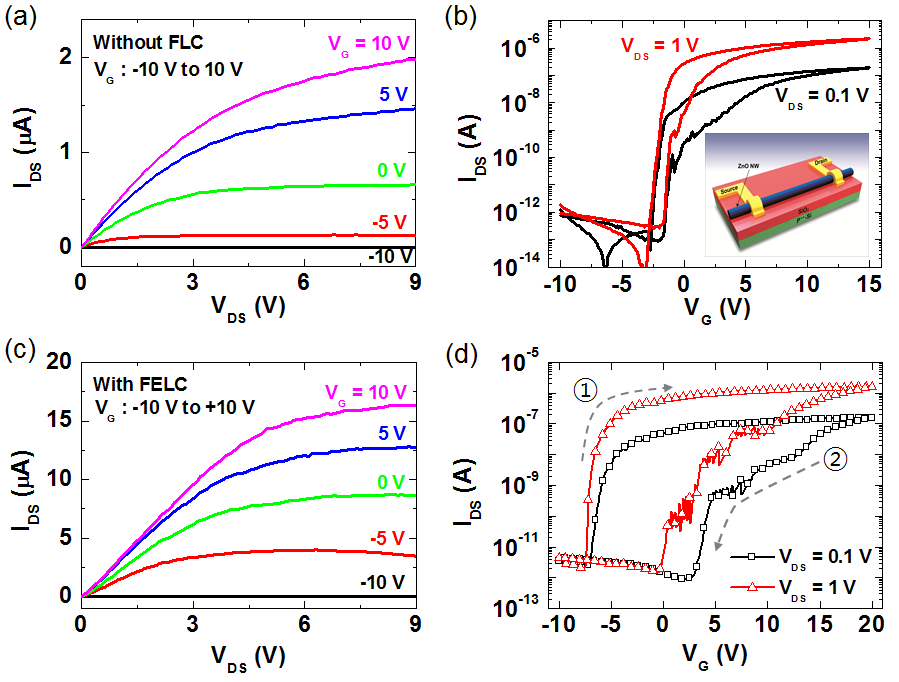
(**a**) Output characteristics (I_DS_-V_DS_) and (**b**) transfer characteristics (I_DS_-V_G_) of a back-gate ZnO nanowire FET without a ferroelectric LC layer. The inset shows a schematic view of a ZnO nanowire FET with a back-gate configuration; (**c**) output characteristics and (**d**) hysteretic behaviours of the ferroelectric LC-coated ZnO nanowire FET. Reprinted with permission from [7]. Copyright 2013, American Institute Physics.

In order to address whether the ferroelectric polarization of LCs can affect the electrical transport in FETs, we performed output (source-drain current *versus* voltage, I_DS_-V_DS_) and transfer (source-drain current *versus* gate voltage, I_DS_-V_G_) measurements with a conventional back-gate FET device structure (the inset of Figure 2b) [7]. Figure 2a,b shows the representative data of the output characteristics and transfer characteristics of a back-gate ZnO nanowire FET without a ferroelectric LC layer, respectively. The output curves exhibit well-defined linear regimes at low biases and saturation regimes at high biases, showing the typical pinch-off characteristics of n-type semiconductor FETs [7,11,12,38]. It was observed that the back-gate nanowire FET exhibited negligible hysteresis, being caused by uncontrolled chemical species adsorbed on the ZnO nanowire when exposed to ambient air [7,12], before coating ferroelectric LCs, as shown in Figure 2b. Figure 2c,d shows the electrical properties of the ferroelectric LC-coated nanowire FET with a back-gate structure. Noticeably, we observed that the electrical performance of ZnO nanowire FETs is significantly improved after coating a ferroelectric layer. Here, note that we performed electrical measurements with the same device before and after the coating of ferroelectric LCs in order to circumvent size and morphology effects, such as the channel length and nanowire diameter. Thus, the main reasons causing the conductance enhancement of the nanowire FET with a ferroelectric LC layer can be due to the polarization effect, as well as the suppression of chemisorption effects of oxygen and/or water molecules at the ZnO surface [7,39]. It was also observed that the FET (Figure 2d) exhibits a clockwise hysteresis behaviour obtained with a double sweep of the gate voltage from −10 to 20 V at variable drain voltages (V_DS_ = 0.1 and 1 V). Here, an interesting finding is that the hysteresis width of the nanowire FET considerably increases after coating a ferroelectric LC layer compared with that of the conventional nanowire FETs without a ferroelectric LC layer (Figure 2b). These results indicate that the channel conductance and threshold voltages of ZnO nanowire FETs are strongly dependent on the channel charge density of the ZnO nanowires, which can be effectively modulated by the polarization of ferroelectric LCs.

**Figure 3.**
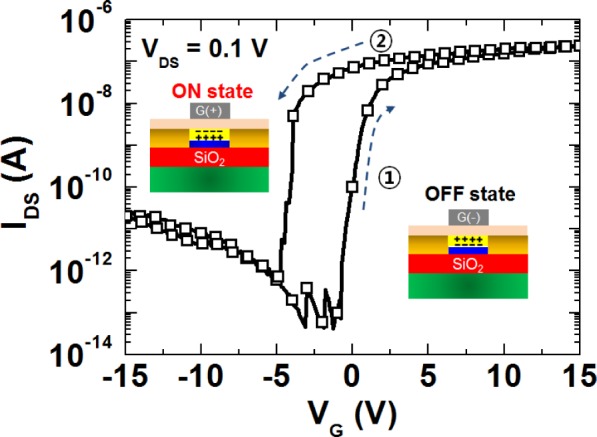
Hysteretic behaviour of a top-gate ZnO nanowire FET with a ferroelectric LC layer.

To further verify the origin of the large hysteresis and modulation of the channel conductance in the nanowire FET device, we fabricated a top-gate nanowire FET (the inset of Figure 3), which is an attractive configuration to allow the favourable polarization of the ferroelectric layer, inducing a more effective field effect on the nanowire channel [7,12]. As expected, we observed that, unlike the hysteresis behaviour of the back-gate nanowire FET with a ferroelectric LC layer, the top-gate nanowire FET with the ferroelectric LC layer exhibits a counter-clockwise hysteresis loop, as shown in Figure 3. That is, in contrast to a back-gate configuration causing the opposite polarization states, as the gate voltage was swept from a negative (positive) value, a negative (positive) polarization of the ferroelectric LC was induced on the nanowire surface. This implies that hysteresis behaviours associated with threshold voltage shifts and channel conductance are strongly dependent on the sign of polarization charges induced in the ZnO nanowire channel according to voltage sweep directions. This is consistent with the results of previous studies on ferroelectric memory transistors [12,40].

**Figure 4.**
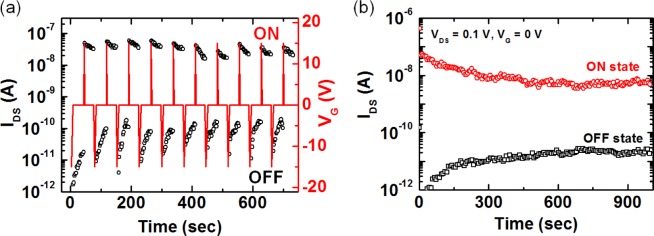
(**a**) Reversible, reproducible ON and OFF switching characteristics of a top-gate nanowire FET with a ferroelectric LC layer; (**b**) retention times for the ON and OFF states of ferroelectric LC-coated FETs measured after the application of a writing pulse (+15 V) and an erasing pulse (−15 V). Reprinted with permission from [7]. Copyright 2013, American Institute Physics.

To demonstrate the practical memory properties of ferroelectric LC-coated nanowire FETs, we investigated the switchability and retention time of a device with a top-gate structure [7]. Figure 4a shows the reversible, reproducible ON and OFF switching characteristics of a memory device. Two different conductance states, defined as the ON (high conductance) and the OFF (low conductance) states of a memory, were measured at the read gate voltage (V_G_ = 0 V) after the application of a writing and an erasing gate voltage pulse of 15 and −15 V, respectively.

Figure 4b shows the retention characteristics of the ferroelectric LC-coated nanowire FET-based memory measured at V_DS_ = 0.1 V and V_G_ = 0 V after a device was switched to the ON and OFF states using writing and erasing gate voltage pulses, respectively. We find that the ON and OFF states were retained over 10^3^ s. Although the retention times of our memory device based on ferroelectric LCs are still short and need to be further improved for data storage device applications, it is noteworthy that we demonstrate proof-of-principle concepts, showing the feasibility of creating a new hybrid system with switch, memory and display functions. Additionally, we believe that the observed memory effects originate from the reversible switching between two polarization states, which are effectively reoriented according to the polarity sign of applied gate voltages and, thus, resulting in the accumulation or depletion of the channel electrons in the ZnO nanowire, as illustrated in Figure 1. This study will provide great potentials for use of LCs in a hybrid system with switch, memory and display functions.

### 2.2. Future Three-Dimensional Holography Based on Liquid Crystals

**Figure 5.**
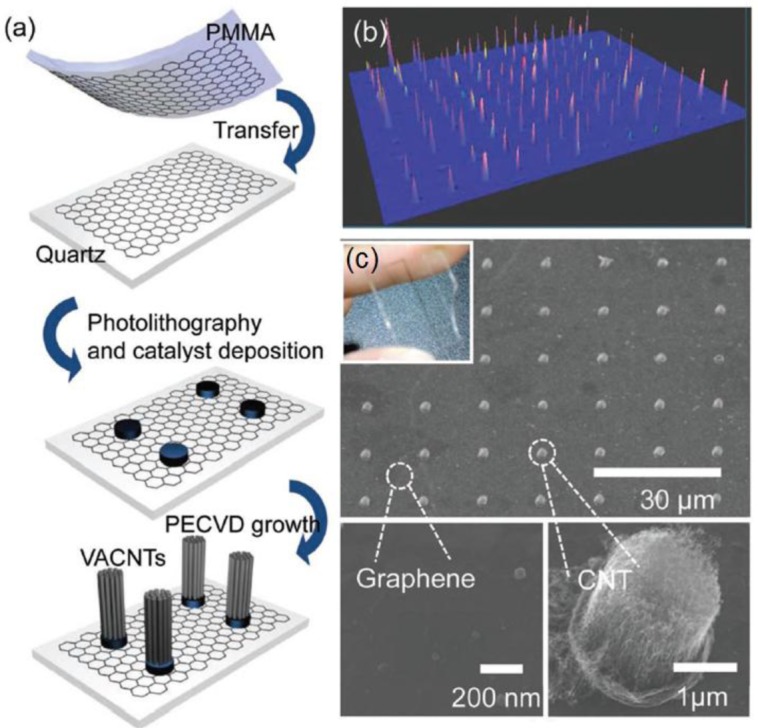
(**a**) Fabrication schematics of graphene/vertically aligned carbon nanotube (CNT) hybrid structure on quartz; (**b**) a 3D image of CNT heights obtained from optical surface profiler; (**c**) scanning electron microscopy (SEM) images of graphene/vertically aligned CNTs on quartz substrates. The inset shows a digital photographic image of our optical element. Reprinted with permission from [16]. Copyright 2011, John Wiley and Sons. VACNT, vertically aligned CNT.

To avoid the requirements of high external voltages to modulate the refractive index of LCs, a combination of a graphene/carbon nanotube (CNT) hybrid structure, such as a transparent active-type 2D optical array, and LCs, as the medium inducing various refractive index changes, can be considered as a viable strategy. An array of CNT pixels was fabricated on the patterned catalysts to modulate the refractive index in a desired location by using chemical vapour deposition, as shown in Figure 5a,c [16]. A strong local field can be generated by geometrically sharp CNTs with a large field enhancement factor, which locally modulates the refractive index of the LC medium at low operation voltages [23–25]. In addition, instead of using opaque metals, graphene with 96% transmittance per layer was used as the transparent electrodes for the transmissive mode for optical devices. Here, note that the height distribution of the CNT forests is important for obtaining uniform diffraction patterns. This distribution was measured using an optical surface profiler. Figure 5b shows that CNTs were uniformly distributed on each pixel with an average height of 1 ± 0.2 μm.

**Figure 6.**
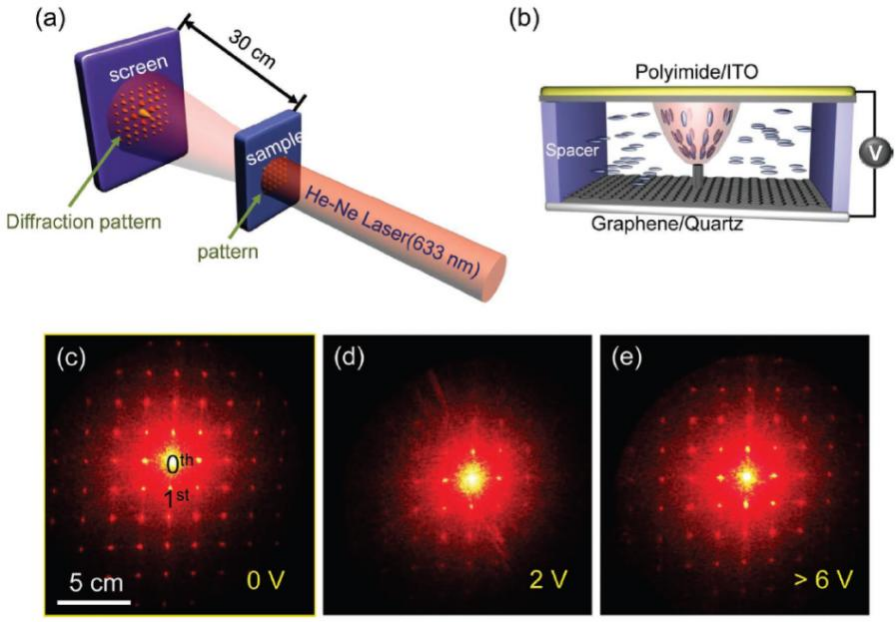
(**a**) Schematic of the measurement for diffraction patterns; (**b**) schematic illustration for the structure of the fabricated optical device. LCs are aligned by the applied electric fields between two electrodes with the spacing of 20 μm. Digital photographic images of diffraction patterns at (**c**) 0 V, (**d**) 2 V, and (**e**) over 6 V, respectively. Reprinted with permission from [16]. Copyright 2011, John Wiley and Sons. ITO, indium-tin-oxide.

Figure 6 shows the diffraction pattern through the graphene/vertically aligned CNTs (VACNTs) hybrid structure as a function of the applied voltage from zero to 6 V [16]. We used a He-Ne Laser with a 633 nm wavelength to generate diffraction patterns from the graphene/VACNT hybrid structure on a quartz substrate, as shown in Figure 6a.

The LC director was aligned randomly in the graphene plane and aligned homogeneously parallel to the upper electrode, whose anchoring was invoked by the rubbing polyimide. Thus, the light can be transmitted partially from the graphene region, whereas CNTs absorb light and emit light again like an antenna, which act as a slit to generate a spherical wave. A series of light transmitted through 2D slits behaves like a 2D Young’s slit, creating a planar interference pattern. LC molecules near the CNTs can be aligned parallel to the long axis of a CNT [41,42], and in particular, those located at the tip of CNTs can be aligned more strongly parallel to the field direction, because the field is strongly enhanced by the sharp CNT tip [43]. The path difference of light from slits can be modulated by the degree of alignment of LC molecules in each slit, being varied by an external electric field. Thus, each pixel acts as a circular slit, and hence, a 2D diffraction pattern from periodically arranged 2D CNT pixels (15 μm space in the current study) was observed, as shown in Figure 6c. As the voltage increases to 2 V, a strong local field enhanced by the CNT tips changes the LC director nearby to modify the local refractive index. Therefore, Δn in the local area increased the change in light intensity of the diffraction pattern, as shown in Figure 6d [23–25,44]. At a voltage higher than 6 V, all the LC directors were aligned along the field direction; therefore, Δn ~ 0, invoking no diffraction pattern changes and, thus, resulting in reducing the light intensity (Figure 6e).

We measured the detailed light intensity changes at the first order spot as a function of voltages for different tip morphologies [16]. As the voltage increases, more LC molecules were aligned along the field, inducing larger Δn near the tip. Thus, this resulted in the increase in the intensity of the first order until a maximum value was reached. After a maximum value of the intensity, the alignment of LC molecules no longer occurs, and therefore, Δn decreases, reducing the intensity of the diffracted first order light. A similar trend was also observed independent of the tip morphology and type of CNTs. We also observed that the peak voltage was reduced when the single-walled CNTs were aligned better, due to a more efficient alignment of LC molecules near the CNTs. The maximum diffraction intensity was observed around 2–3 V. As expected, a higher peak voltage was required for multi-walled CNTs due to their poor alignment and/or smaller field enhancement factor [41]. It is further noted that the degree of alignment of the CNTs plays an important role in improving the diffraction light intensity. We believe that our current approach for the development of transparent optical elements could be beneficial for realizing future 3D holography.

### 2.3. Self-Powered Liquid Crystal Displays

In addition to innovative and conceptual approaches to future LC applications, practical and multidisciplinary applications via the convergence of displays and energy generation technology have also attracted considerable attention. There is no doubt that the mobile information technology is one of the most important key features describing current society. In particular, display technology represented by LC displays in mobile devices is an essential, commonly used component for the information exchanges.

One of the most important issues in mobile devices is sustainable energy supply. Although the battery technology has been continuously improved, so far, it has always been required to realize sustainable cord-free, *i.e.*, self-powered devices. For this reason, various energy harvesting technologies have been actively studied, especially, and piezoelectric nano-energy harvesting has been considered as a promising technology, which is accelerated with the development of nanotechnology.

**Figure 7.**
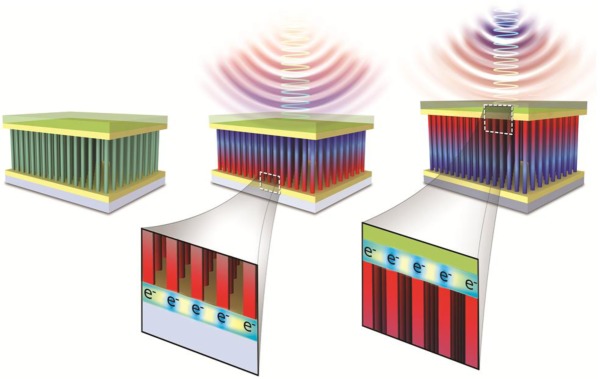
Operation principle of a piezoelectric energy harvester fabricated with ZnO nanowires. Reprinted with permission from [26]. Copyright 2013, Royal Society of Chemistry.

The energy harvesting functionality of piezoelectric ZnO nanowires arises from the modulation of the piezoelectric polarization when the mechanical force is applied on the nanowire, which induces the relative displacement of the Zn cations with respect to the O anions along the strain direction [33]. This piezoelectric effect produces ionic charges distributed along the nanowire and, hence, results in the piezoelectric field, which drives electrons to flow in an external circuit to balance the piezoelectric potential generated across the two ends of a nanowire. Consequently, the negative piezoelectric potential developed on the compressed top side of a nanowire by mechanical stress leads to the accumulation of electrons on the bottom side (the middle of Figure 7), whereas when the external force is released, the accumulated electrons on the bottom side flow back to the top side (the right of Figure 7) through the external circuit in order to balance the difference in Fermi levels at the two sides [26].

**Figure 8.**
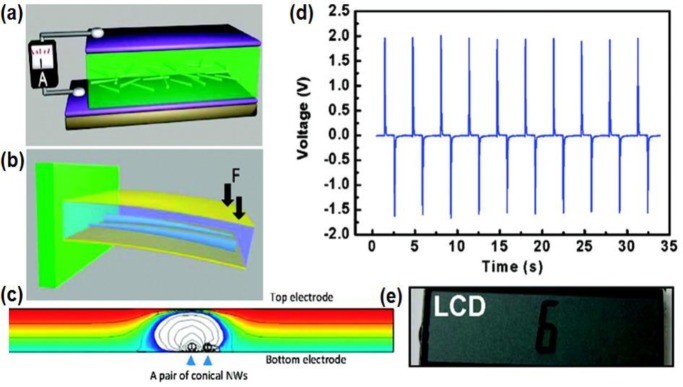
(**a**) A schematic image of the fabricated energy harvester using ZnO conical nanowires (CNWs) and (**b**) a model showing the setup for measuring the energy conversion. The polystyrene substrate used to hold the nanogenerator at its upper side, where the force, F, is applied. The CNWs are under compressive strain during the deformation; (**c**) the model used for calculating the potential distribution across the top and bottom electrodes of the harvester with the presence of a pair of CNWs; (**d**) the measured output voltage; (**e**) a snap shot of an LC display driven by the harvester. Reprinted with permission from [45]. Copyright 2010, American Chemical Society.

LCs, basically, are operated by voltages, which are favourable power consumption characteristics to the piezoelectric energy harvester without the need for a high current supply. In this regard, Wang’s group at the Georgia Institute of Technology reported that a piezoelectric nano-generator can generate enough energy to drive a small LC display (Figure 8) [45]. They demonstrated that the harvesting performance is enhanced by using conical ZnO nanowires. Due to the geometrical shape, the nanowires are assembled with a unipolar feature, which is probably the key for producing a macroscopic piezoelectric potential in the direction normal to the substrate. This harvester produced output voltage up to 2 V (an equivalent open circuit voltage of 3.3 V) with a few hundreds of nano amperes of output current, which can drive a small LCD panel.

For sustainable piezoelectric energy harvesting to drive integrated devices, such as LC displays, it is important to target the proper mechanical energy sources. Sound is one of the most common and abundant mechanical energies in our daily life. However, due to the rarefaction nature of the air, the media of sonic waves, it is difficult to harvest the mechanical energy of sound. Since the first sonic-driven piezoelectric energy generator was reported by using of ZnO nanowires in 2008 [30], various efforts have been continued to improve the performance of sound energy harvesters. As a result, recently, it has been reported that enhanced sound energy harvesters can produce enough output power to operate a polymer-dispersed LC (PDLC) light shutter [27,46,47].

**Figure 9.**
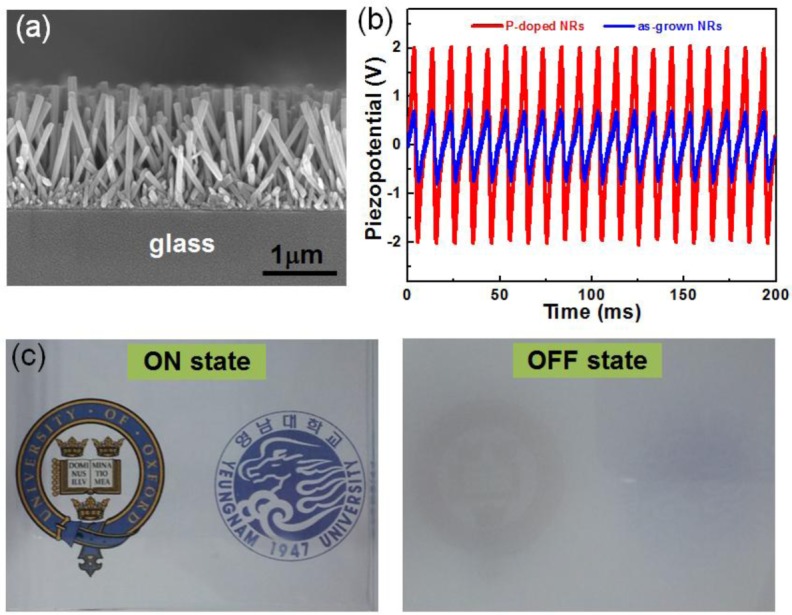
(**a**) SEM images of p-type ZnO:P nanorods on a glass substrate; (**b**) the piezoelectric output potential measured from sound-driven piezoelectric nanogenerators (SPNGs) consisting of ITO-coated flexible polyethylene terephthalate (PET) as a top electrode and ZnO nanorods before and after P-doping; (**c**) a polymer-dispersed LC (PDLC) device (4 cm × 5 cm) switched on (**left**) by an SPNG and (**right**) before being switched on using an SPNG, respectively. Reprinted with permission from [27]. Copyright 2014, Royal Society of Chemistry.

Recently, we successfully demonstrated operating PDLC light shutters using sonic-driven piezoelectric energy generators, as shown in Figure 9 [27]. It has been well known that the free electrons in an as-grown ZnO nanowire significantly screen piezoelectrically generated charges, resulting in decreasing the output piezopotential [26]. Therefore, to enhance the output characteristics, we introduced p-type counter dopants, such as P and Ag, to eliminate the free carrier screening effect by decreasing free electrons in the ZnO nanowire, because as-grown ZnO nanowires are naturally n-type semiconducting with superior conductivity due to oxygen deficiencies [26]. The doping process was carried out at low temperature compatible with glass substrate-based processes by using all solution processes based on a spin-on-dopant method [27]. Here, it is important to note that the low temperature process is crucial for the hybrid system to integrate energy harvesters into displays, because most of all the display components are fabricated based on glass or plastic substrates.

**Figure 10.**
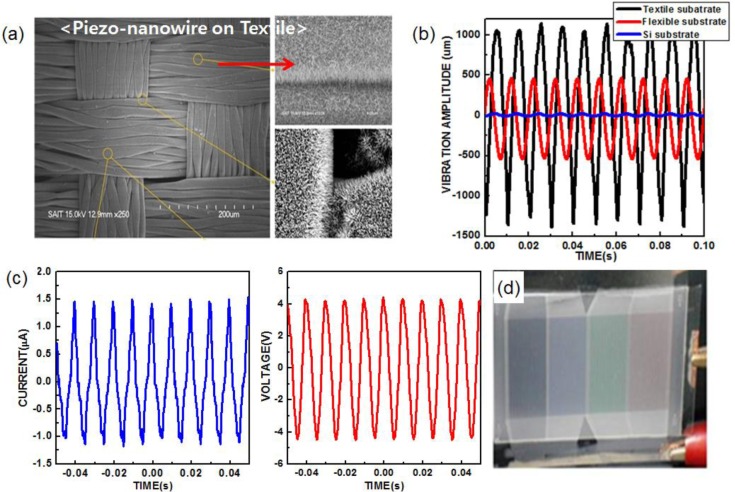
(**a**) Large-area SEM images of ZnO nanowires grown on a textile substrate; (**b**) comparison of the vibration amplitude of the Si, flexible PES and textile substrates that were vibrated by a sonic wave; (**c**) output voltage and current from the large-area textile harvester (10 cm^2^) at 100 dB and 100 Hz; (**d**) a PDLC display panel operated by the sound-driven textile harvester. Reprinted with permission from [46]. Copyright 2012, Royal Society of Chemistry.

Furthermore, in order to address the issues related to the mechanical impedance of the substrate, we investigated various substrates toward an optimized harvesting system, as external mechanical energy sources are generally transferred into piezo-structures through the substrate. In this regards, textile with favourable form factors is one of the promising substrates for an effective harvester, due to its wide degree of freedom in shapes with flexible and stretchable characteristics, as well as higher efficiency in coupling external sonic waves into its own vibrational motion (Figure 10). Figure 10a shows the vibrational amplitudes of three different substrates, Si, flexible polyethersulfone (PES), and textile measured under the same input sonic power of 100 dB at 100 Hz using a laser optical vibrometer. The vibrational amplitudes were 30, 960 and 2250 μm for Si, flexible PES and textile substrates, respectively. With the optimized structures based on a textile substrate, the high output voltage and current of the harvester (8 V and 2.5 μA from 10 cm^2^) were generated as shown in Figure 10c. In addition, to demonstrate the capability of directly driving a practical display device, reflective mode display (PDLC display) panels (9 cm × 3 cm) were driven by the textile nanogenerator (Figure 10d) [46].

**Figure 11.**
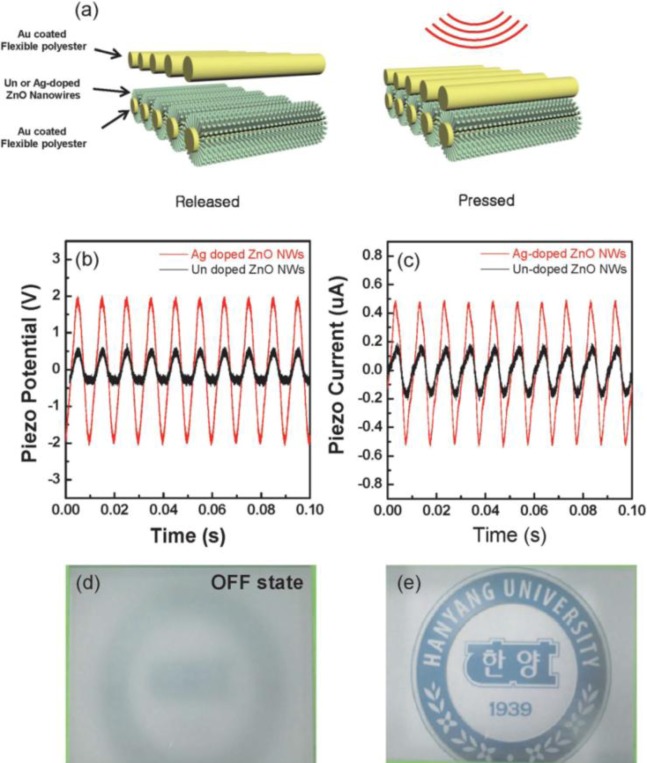
(**a**) Schematic diagram of device operation for the ZnO nanowire-based nanogenerator grown on the flexible polyester; (**b**) piezoelectric potential and (**c**) piezoelectric current measured from Ag-doped ZnO nanowires (red line) and un-doped ZnO nanowires, respectively, (black line) on polyester substrates using sound waves; (**d**) OFF-state of the PDLC device; (**e**) ON-state of the PDLC device after generating sound waves. Reprinted with permission from [47]. Copyright 2013 Royal Society of Chemistry.

It is also expected that the combination of carrier controlled ZnO nanowires and textile substrates would be one of the viable ways to generate higher output power. To this end, we directly synthesized Ag-doped ZnO nanowires by using a hydrothermal synthesis method fabricated under 90 °C without introducing any high temperature post-annealing processes. The Ag-doped nanogenerators made based on 10 cm^2^ of a polyester textile substrate generated a power of 0.5 μW under a sound input of 80 dB, 100 Hz, which is nearly 2.9 times higher than that of a nanogenerator with un-doped ZnO nanowires, and successfully operated PDLC light shutters (Figure 11) [47]. These findings imply that our doping approach at low temperature provides opportunities for the potential use of textile nanogenerators with high performance as self-powered devices.

Grid-free displays, especially powered by an energy harvester, will be the basis serving as a key component for future mobile devices, and our study on the piezoelectric energy generator integrated with LC displays will provide great potential for realizing grid-free display systems.

## 3. Experimental Section

### Growth of ZnO nanowire arrays

ZnO nanowires were grown on a-plane sapphire substrates with Au catalysts by a vapour transport method. ZnO powder and graphite powder were mixed in a weight ratio of 1:1, and then, the mixed powders were ground together. A layer of Au thin film (~3 nm) was deposited on the sapphire substrate using electron-beam evaporation. The source materials and substrates were placed in an alumina boat, which was loaded at the centre of an alumina reaction tube located in a high-temperature tube furnace. The ZnO nanowires were then grown on the substrates at a temperature of 930 °C for 20 min under a flow of Ar (23 sccm [standard cubic centimeters per minute]).

### Fabrication of nanowire FETs

To fabricate ZnO nanowire FET devices, ZnO nanowires were first dispersed by sonication in isopropyl alcohol and then transferred onto a silicon substrate by dropping a liquid suspension of ZnO nanowires from a pipette. A 100 nm-thick silicon oxide layer was employed as a gate oxide layer on a heavily doped p-type silicon substrate used as a global back gate. Source and drain electrode patterns were defined by conventional photolithography followed by electron beam evaporation of Ti (80 nm)/Au (40 nm) electrodes on a nanowire and lift off processes. The separation of source and drain electrodes was ~3 μm. The ferroelectric LC was simply spin-coated at 4000 rpm onto the ZnO nanowire channel by using a solution-based spin-coating method. For the top-gate nanowire FETs, cross-linked poly(4-vinylphenol) (PVP) (acting as a dielectric layer) was coated on the prepared substrate with source and drain electrodes, followed by a curing step. The cross-linked PVP dielectric layer was prepared by mixing PVP (10 wt% of solvent) with additive poly(melamine-co-formaldehyde) methylated (5 wt% of solvent) in propylene glycol monomethyl ether acetate (PGMEA). Top-gate electrodes were made by depositing a 50 nm-thick Au layer on the PVP layer using the electron beam evaporator.

### Graphene/VACNT hybrid structure

Graphene was synthesized on a Cu foil (75 μm) using a thermal chemical vapour deposition (CVD) method and transferred onto the quartz substrate. The reaction chamber was heated to 1000 °C and then CH_4_ (5 sccm), Ar (1000 sccm) and H_2_ (200 sccm) gases were introduced into the reaction chamber for 2 min. After coating PMMA film on a graphene/Cu substrate, the Cu film was removed by submerging the film into Cu etchant (CE-100, TRANSENE Company, Danvers, MA, USA). The graphene/PMMA film was then transferred onto a quartz substrate. This process was repeated to obtain several layers of graphene. Conventional photolithography was used to make dot arrays with a ~1 μm dot size and ~15 μm spacing between dots. 10 nm Al and 1 nm Fe layers were deposited as catalysts for CNT growth. Vertically aligned CNTs were synthesized by remote plasma-enhanced CVD with Ar (200 sccm), H_2_ (200 sccm) and C_2_H_4_ (75 sccm) gases at 750 °C for 5 min.

### Optical cell assembly

The graphene/VACNTs hybrid structure was covered with the top ITO glass electrode separated by a 20-μm spacer. ITO glass was coated with polyimide and rubbed for horizontal alignment of the liquid crystal. A nematic liquid crystal with positive dielectric anisotropy (Merck, Darmstadt, Germany, ZLI 4792) was injected into the cell.

### Polymer dispersed liquid crystal

PDLC composite materials consisting of a nematic LC TL203 (Merck, the ordinary refractive index no = 1.529) and a monomer PN393 (Merck, the polymer refractive index np = 1.499) with weight ratio of 78:22 has been made by applying a UV-polymerization-induced phase separation method. The intensity of the UV light for curing the monomers was 20 mJ/cm^2^, and indium-tin-oxide (ITO)-coated glass was used as the device substrates.

## 4. Conclusions

We present recent research snapshots of LCs that are focused on developing new LC applications. We recently demonstrated emerging applications of LCs ranging from electronics to holography and self-powered systems. Moreover, we describe novel concepts and operating principles of new memory and transparent and active-type 2D optical array devices based on LCs. Finally, we show self-powered display systems integrated with piezoelectric nanogenerators as one of the key technologies for future grid-free displays. This review will give insights not only into understanding the basic technical and scientific applications behind LCs, but also into developing new discoveries of the practically use of other LC-based devices.
